# From Kidney Crisis to Heart Revival: A Case of Septic Cardiomyopathy Following Septic Shock Induced by Xanthogranulomatous Pyelonephritis

**DOI:** 10.7759/cureus.72560

**Published:** 2024-10-28

**Authors:** Abderrahmane Bouchaala, Chadi Bourimi

**Affiliations:** 1 Cardiology, University Hospital Ibn Sina, Rabat, MAR; 2 Urology A, University Hospital Ibn Sina, Rabat, MAR

**Keywords:** left ventricle dysfunction, sepsis, septic cardiomyopathy, septic shock, xanthogranulomatous pyelonephritis

## Abstract

Septic cardiomyopathy (SCM) is a reversible cardiac dysfunction occurring in patients with sepsis or septic shock, characterized by transient impaired myocardial contractility. Despite its prevalence in severe sepsis and significant impact on patient outcomes, SCM remains underdiagnosed and is one of the least studied forms of cardiomyopathy. Its recognition and management are further complicated by the absence of a universally accepted consensus on its diagnostic criteria and definition. We report the case of a 54-year-old female presenting with septic shock secondary to xanthogranulomatous pyelonephritis (XGP). The patient presented with acute right lower back pain, fever, and hemodynamic instability. Initial echocardiography assessment demonstrated a preserved left ventricular ejection fraction (LVEF), which subsequently deteriorated following the onset of septic cardiomyopathy. Early recognition and management, including the use of vasopressors, inotropes, and judicious fluid resuscitation, resulted in full recovery of cardiac function within one week. This case highlights the importance of clinical vigilance and prompt intervention in the management of SCM, particularly in cases complicating severe infections such as XGP.

## Introduction

Septic Cardiomyopathy, known also as sepsis-induced cardiomyopathy, or sepsis-induced myocardial dysfunction, can be defined as a sepsis-induced acute cardiac dysfunction of non-ischemic origin, manifesting as varying degrees of systolic and/or diastolic dysfunction involving either or both the left and right ventricles [1]. Despite the absence of consensual definition of septic cardiomyopathy, most scholars agree on few essential criteria: acute ventricular dysfunction (systolic and/or diastolic), reversible within 7 to ten days, with reduced response to fluid resuscitation and catecholamines and the absence of acute coronary syndrome [2,3]. While mortality in patients presenting septic shock exceeds 40%, the presence of septic cardiomyopathy in septic shock reportedly increases the mortality rate in patients by 70% to 90% [3-5].

Xanthogranulomatous pyelonephritis is a rare yet destructive form of pyelonephritis, classically caused by chronic infection and nephrolithiasis. This variant is most frequently associated with chronic obstruction and urinary tract calculi with ongoing or chronic infection [6]. Early diagnosis and prompt management are essential in reducing morbi-mortality as well as preventing severe complications, particularly septic shock [7]. In this report, we present a case of sepsis-induced cardiomyopathy following septic shock secondary to acute xanthogranulomatous pyelonephritis.

## Case presentation

A 54-year-old female initially presented to the emergency department with signs of dizziness, acute right lower back pain, and fever. Two days prior to admission, the patient reported dysuria accompanied by pyuria and a fever of 39°C. On admission, a clinical examination found a somnolent patient (Glasgow Coma Scale of 14), febrile (body core temperature of 38.6°C), polypneic at 25 breaths per minute with room air saturation SpO_2_:97%. Her blood pressure was 92/48 mmHg, with signs of peripheral hypoperfusion. Abdominal examination revealed a palpable mass in the lumbar region with tenderness on palpation, while cardiopulmonary and arterial auscultations were unremarkable.

**Figure 1:**
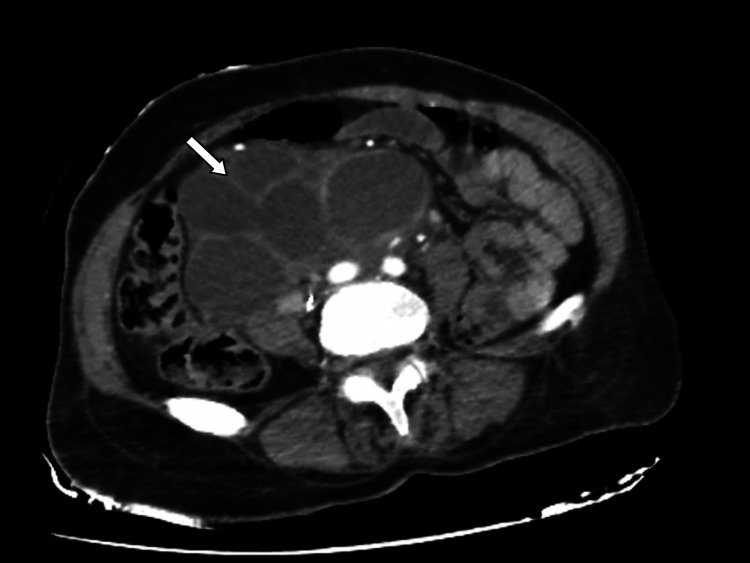
Contrast-enhanced CT scan of the abdomen and the pelvis (axial view) Axial contrast-enhanced computed tomography of the abdomen and the pelvis showing a giant ectopic right kidney (17.7 cm × 13.5 cm), dilated renal pelvis and calyces with multiple central hypodense areas, round in shape, enclosed by an enhancing rim of contrast and peripheral fat stranding (white arrow).

Abdominal computed tomography revealed an ectopic, enlarged right kidney exhibiting multiple rounded hypodense areas encircled by an enhancing rim of contrast alongside significant pyelocaliceal dilation, and peripheric fat stranding (Figure 1). The left kidney appeared normal in location, size, and morphology, leading to the diagnosis of shock secondary to xanthogranulomatous pyelonephritis.

The patient was stabilized with fluid expansion treatment, norepinephrine administration at 0.5 μg/kg/min, and broad-spectrum antibiotics: ceftriaxone 2 g/day and amikacin 1 g/day. Urgent surgical intervention was performed, involving the placement of an internal double-J stent for drainage. Perioperative cytobacteriological analysis of the pus revealed *E. coli*, which was sensitive to the empiric antibiotic regimen already in place.

**Figure 2:**
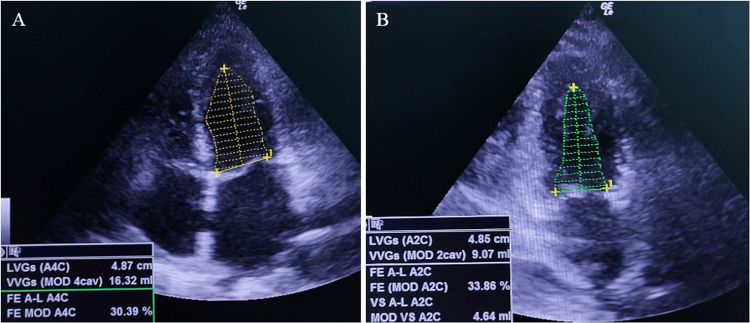
Transthoracic echocardiography was performed on the third postoperative day (A) Four-chamber view demonstrating severe left ventricular dysfunction with markedly reduced contractility across all segments of the left ventricle. (B) The two-chamber view, providing a detailed assessment of the left ventricle, further highlighting the depressed myocardial function. LVEF: 32%. LVEF: left ventricle ejection fraction.

On the second postoperative day, the patient exhibited improved blood pressure control, decreased norepinephrine requirements, fever, and pain relief, along with a reduction in C-reactive protein (CRP) and other inflammatory markers. However, on the third day, the patient experienced a sudden drop in blood pressure; trans-thoracic echocardiography demonstrated a significant deterioration in systolic function compared with the initial echocardiography (Figure 2).

**Figure 3:**
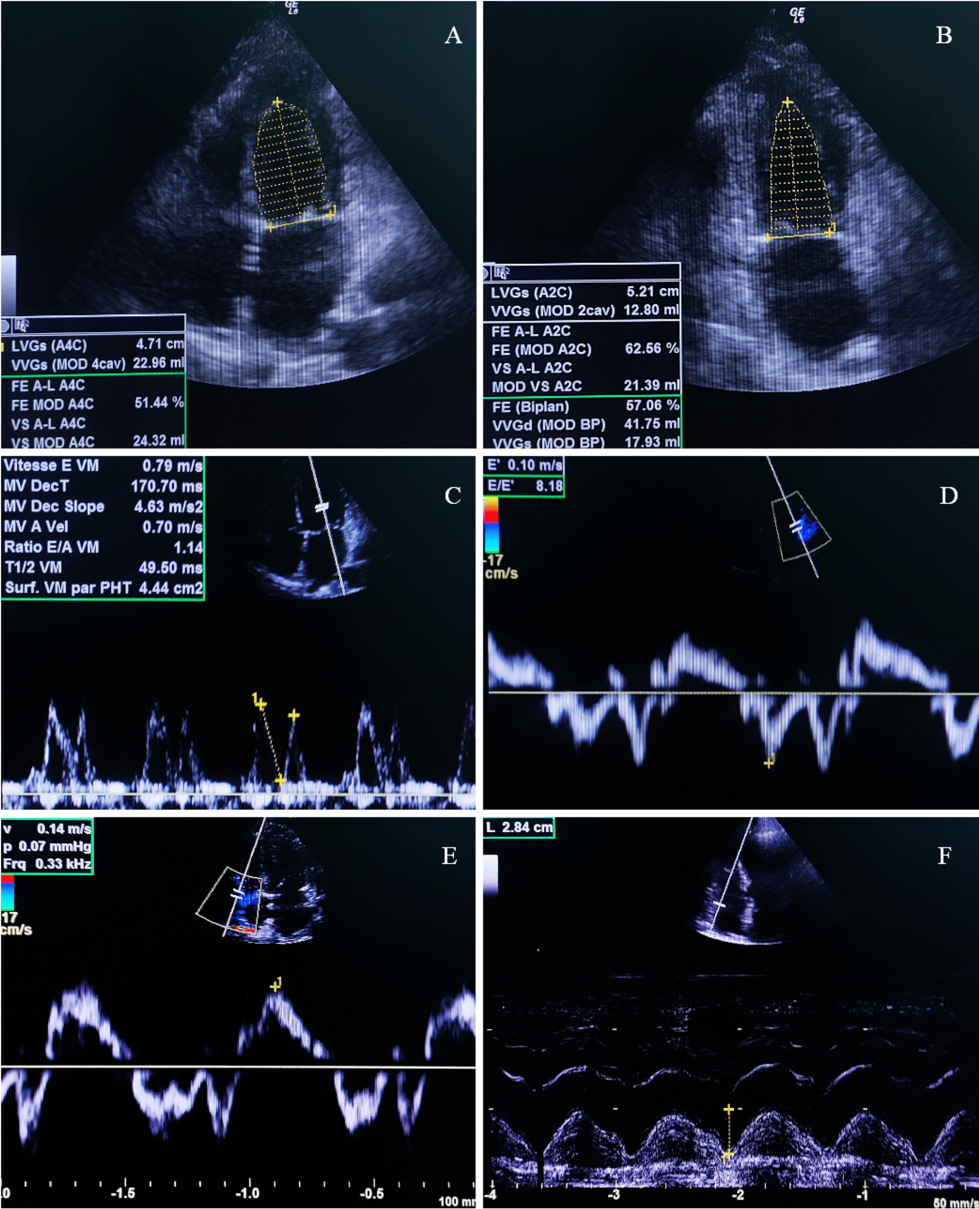
Transthoracic echocardiography performed on the seventh postoperative day (A) Four-chamber view in bidimensional mode and (B) two-chamber view mode both show complete normalization of left ventricular systolic function, LVEF: 57%. (C) Pulse-wave Doppler on the mitral funnel showing a normal transmitral filling pattern (E:79 cm/s, A:70cm/s E/A:1.14). (D) Tissue Doppler Imaging (TDI) at the lateral annulus, demonstrating normal left ventricular filling pressures, lateral E/e' ratio of 8. (E) TDI of the basal part of the right ventricle free wall, showing a normal systolic velocity S’ of 14 cm/s. (F) M-mode image illustrating a normal tricuspid annular plane systolic excursion (TAPSE) of 28 mm. LVEF: left ventricle ejection fraction.

The patient was managed with inotropic support, combining dobutamine and norepinephrine. Notably, cardiac function normalized within one week, and the patient made a full recovery. Follow-up echocardiography confirmed the resolution of septic cardiomyopathy (Figure 3).

## Discussion

Septic cardiomyopathy is an underdiagnosed complication of severe infections and a significant contributor to cardiovascular collapse in patients experiencing severe sepsis and septic shock. It represents an aspect of the multi-organ failure observed in advanced septic states [5,8]. Given the absence of a consensus regarding the definition or diagnostic criteria, the diagnosis of septic cardiomyopathy can be challenging. Echocardiography remains the gold standard for diagnosing SCM and, by extension, its defining characteristics, including left ventricular systolic dysfunction, as evidenced by depressed left ventricular ejection fraction and global longitudinal strain (GLS), along with left ventricular diastolic dysfunction, reflected by altered Tissue Doppler Imaging TDI-derived septal e' wave velocities and an E/e' ratio. Right ventricular dysfunction may also be present, Notably, these abnormalities typically present suddenly and are often reversible within 10 days, in the absence of coronary artery disease or any alternative cardiomyopathy that could explain these findings [1,2,9].

Septic cardiomyopathy is a complex condition resulting from multiple factors, leading ultimately to myocardial dysfunction during severe sepsis. Central to its pathophysiology is the dysregulated inflammatory response, with main myocardial depressant factors including cytokines such as interleukin (IL)-6, tumor necrosis factor-alpha (TNF-α), and IL-1β, along with nitric oxide dysregulation and components of the complement system [5,10,11]. These mediators contribute significantly to mitochondrial dysfunction, abnormal calcium movements, autonomic dysregulation, and myocyte apoptosis [2,12]. Additionally, the imbalance between pro-inflammatory and anti-inflammatory cytokines disturbs the endothelial glycocalyx, further contributing to vascular injury and heterogeneous microvascular flow, leading to myocardial edema, which recent studies suggest may be an underexplored mechanism of SCM [13].

In early studies, septic cardiomyopathy patients with reduced left ventricular ejection fraction (LVEF) and lower cardiac index were found to have lower mortality rates [14,15], yet subsequent research on the prognostic significance of LVEF yielded conflicting results, highlighting its limited predictive value [2,16,17]. Recent investigations suggest that altered myocardial strain, assessed by echocardiographic speckle tracking, is a more reliable predictor of increased mortality in septic cardiomyopathy. Additionally, isolated right ventricular dysfunction has been associated with worse long-term survival outcomes [1,2].

Xanthogranulomatous pyelonephritis is a rare chronic inflammatory disorder of the kidney, characterized by the destruction of renal parenchyma and its replacement by foamy macrophages and granulomatous tissue. The condition is primarily associated with chronic urinary tract obstruction, most commonly due to nephrolithiasis or obstructive uropathies, whether congenital or acquired [6,18]. While urosepsis and septic shock are redoubtable complications, our case report is unique in the literature for highlighting the diagnostic and management challenges posed by the rare combination of xanthogranulomatous pyelonephritis and septic shock leading to septic-induced cardiomyopathy. The initial preoperative echocardiographic assessment was pivotal in establishing a baseline LVEF, providing a valuable reference as septic cardiomyopathy progressed. Early detection of cardiac involvement in septic shock was essential as it directly impacts therapeutic strategies: usage of both dobutamine and norepinephrine, and restricting fluid expansion, which led to favorable outcomes in our patient. Although large-scale studies on the management of septic cardiomyopathy remain limited, standard therapeutic approaches typically involve early initiation of vasopressors, inotropes, and judicious fluid resuscitation [2,10]. In cases of refractory sepsis-induced cardiogenic shock, early implementation of veno-arterial extracorporeal membrane oxygenation (VA-ECMO) has been shown to be beneficial, yet without larger studies, mechanical support should be considered in extreme cases [2,5,19].

## Conclusions

This case highlights the importance of recognizing septic-induced cardiomyopathy as a potential complication in patients with severe sepsis, particularly when acute hemodynamic deterioration occurs despite improvement in the infectious process. Our patient presented with an exacerbation of xanthogranulomatous pyelonephritis, a severe form of chronic pyelonephritis known to predispose to all septic complications, including septic cardiomyopathy. Although rare, the association between these conditions demands heightened clinical vigilance, underscoring the need for early and frequent echocardiographic monitoring, alongside aggressive supportive care to optimize the outcomes in similar cases.
